# Anti-p200 pemphigoid with autoantibodies targeting laminin beta-4 and clinical response to methotrexate

**DOI:** 10.1016/j.jdcr.2025.02.051

**Published:** 2025-07-28

**Authors:** Mia Siebmanns, Pauline Bernard, Olesya Pavlova, Enno Schmidt, Winfried A. Stöcker, Emmanuella Guenova

**Affiliations:** a Department of Dermatology, Lausanne University Hospital CHUV, University of Lausanne, Lausanne, Switzerland; b Department of Dermatology, University of Lübeck, Lübeck, Germany; c Clinical and Immunological Laboratory Stöcker, Groß Grönau, Germany; d Medical Faculty, Clinical Department for Immunodermatology, Johannes Kepler University, Linz, Austria; e Medical Faculty, Clinical Research Institute for Inflammatory Medicine, Johannes Kepler University, Linz, Austria

**Keywords:** anti-p200 pemphigoid, autoimmune blistering diseases, laminin beta-4

## Introduction

Anti-p200 pemphigoid is a subepidermal autoimmune bullous disease characterized by autoreactive antibodies targeting structural components of the dermoepidermal junction. Most patients present with itchy plaques and tense vesicles or blisters of the skin and the mucous membranes. It can result in sepsis and death in severe cases.^1^ Anti-p200 pemphigoid owes its name to the reactivity of antibodies to a 200 kDa protein of the dermoepidermal junction detected in the extract of human dermis.^2^ Subsequently, laminin γ1 was described as a target antigen recognized in 70% to 90% of cases.^3^ Recently, a new type of antigen has been identified as the target of anti-p200: the laminin β4 subunit in the cutaneous basement membrane zone (BMZ).^4^

We report a case of an anti-p200 pemphigoid with autoantibodies against laminin β4 and responding to a combined treatment of prednisone and methotrexate.

## Case report

An 85-year-old woman with no personal or family history of dermatological diseases presented with a 1-week history of multiple pruriginous papular skin lesions with central excoriations, including her palms, acutely growing in number. Despite treatment with 0.3 mg/kg/day of oral prednisone, her symptoms worsened with chills, general malaise, and oral discomfort.

**Fig 1:**
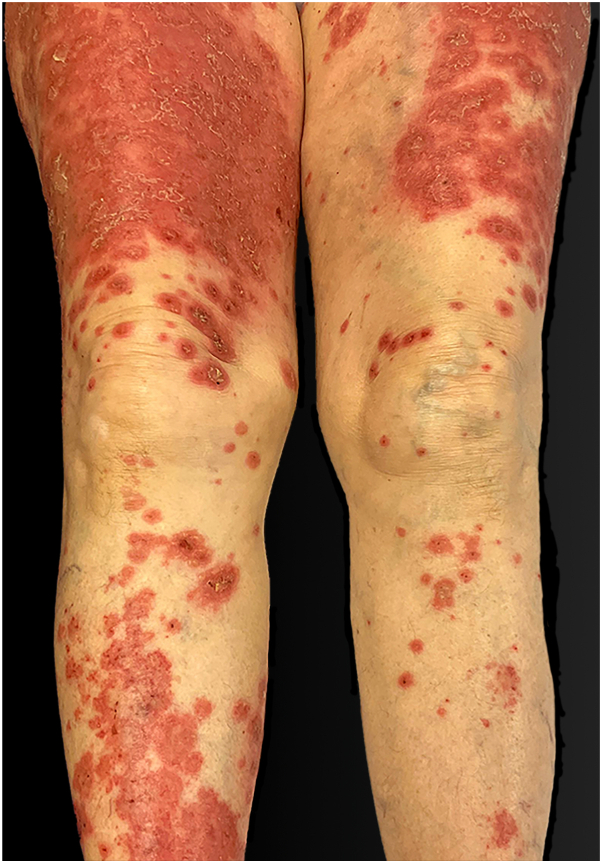
Anti-p200 pemphigoid, initial clinical patient presentation. Legs of the patient. Notice tense and ruptured blisters over erythematous and oedematous skin.

Clinical examination showed a few target-like lesions and tense blisters over well-defined confluent erythematous plaques over the trunk, limbs, palms, and soles (Fig 1). Mucous membranes were involved on the right jugal mucosa and vulva. This clinical picture was suspicious for bullous pemphigoid, with a bullous pemphigoid disease area index score of 58/360.

**Fig 2:**
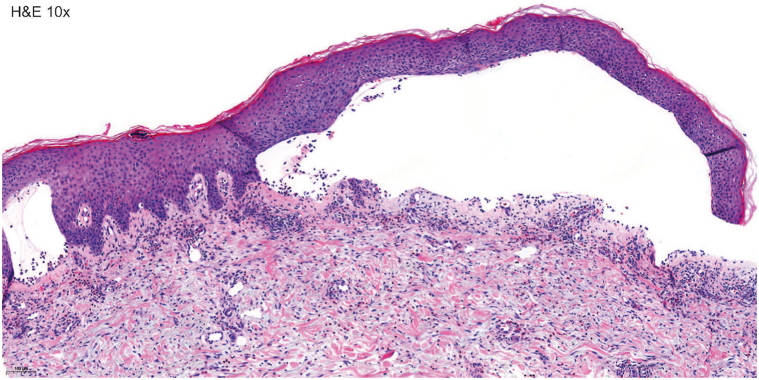
Anti-p200 pemphigoid, hematoxylin & eosin (H&E) staining shows the eosinophilic spongiosis with a subepidermal bulla and mixed lymphohistiocytic infiltrate of the superficial dermis with numerous eosinophils. Scale bars = 100 μm.

Histological examination of a lesional biopsy taken on the edge of a bulla on the left thigh showed an eosinophilic spongiosis with a subepidermal bulla formation and a mixed lymphohistiocytic infiltrate of the underlying dermis with numerous eosinophils, overall consistent with a pemphigoid disorder (Fig 2).

**Fig 3:**
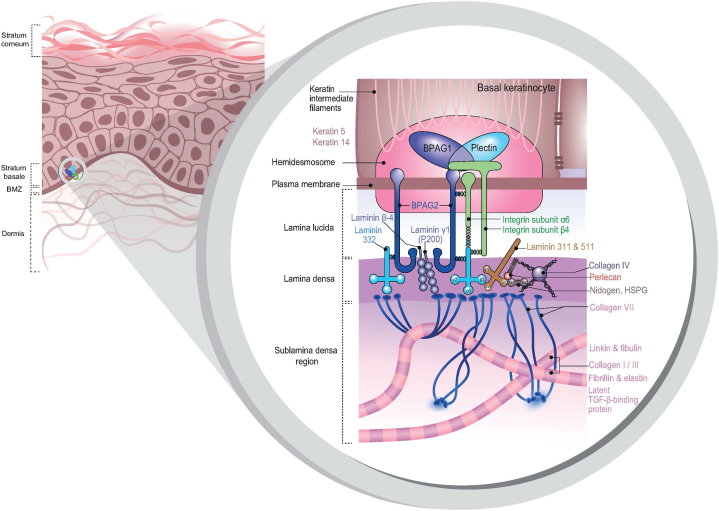
Schematic representation of the components of the basal membrane zone.

The diagnosis of autoimmune blistering diseases involves the detection of autoantibodies against various antigens (Fig 3). The gold standard for the precise diagnosis is the integration of direct and indirect immunofluorescence (IF) alongside additional serological tests, such as enzyme-linked immunosorbent assays.^5^

**Fig 4:**
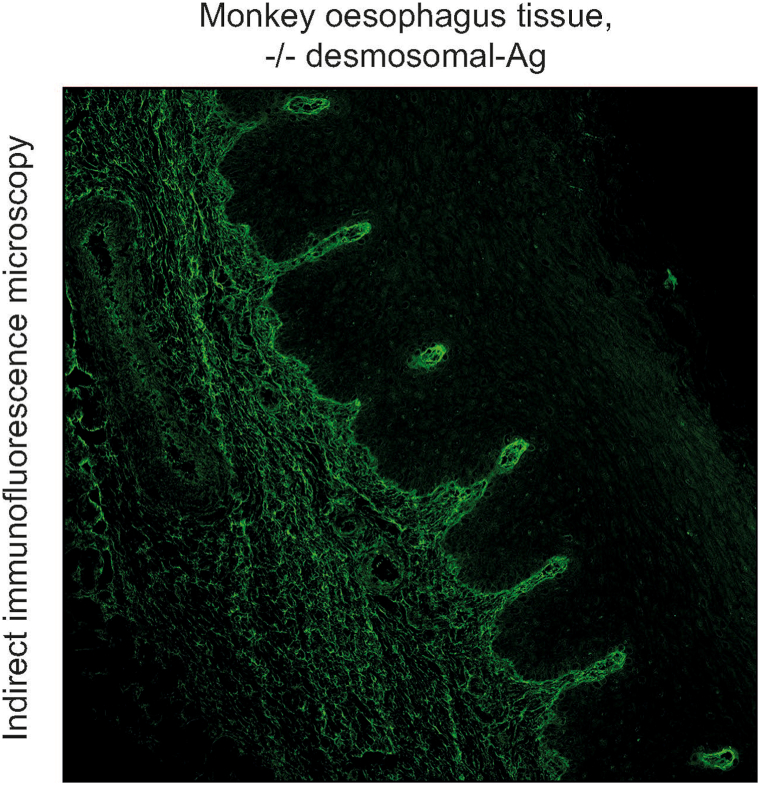
Anti-p200 pemphigoid, indirect immunofluorescence microscopy (BIOCHIP/EUROPLUS mosaics) showing negativity for antibodies against desmosomal antigens on monkey oesophagus tissue.

**Fig 5:**
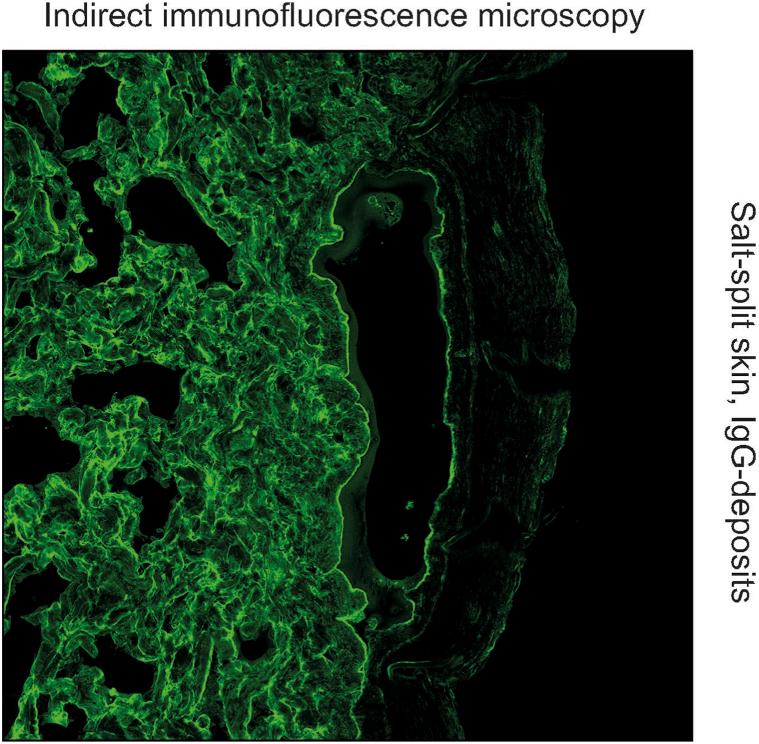
Anti-p200 pemphigoid, indirect immunofluorescence microscopy (BIOCHIP/EUROPLUS mosaics) shows a reactive bulla based on salt-split skin with deposits of IgG antibodies. *IgG*, Immunoglobulin G.

In our patient, the direct IF revealed linear deposits of immunoglobulin (Ig) G and C3 along the dermoepidermal junction. By indirect IF on the monkey oesophagus, neither IgG nor IgA against desmosomal antigens could be detected (Fig 4). Indirect IF on human salt-split skin showed linear binding of IgG along the blister floor compatible with reactivity against type VII collagen, laminin 332 or the p200 antigen as seen in epidermolysis bullosa acquisita, anti-laminin 332 mucous membrane pemphigoid, and anti-p200 pemphigoid, respectively^6^ (Fig 5).

The primary antigens associated with pemphigoid diseases are BP180 (collagen XVII) and BP230. However, the BIOCHIP analysis (Dermatology Mosaic 7) revealed nonreactivity to BP180 and BP230 and Desmoglein 1 and Desmoglein 3. Enzyme-linked immunosorbent assay results confirmed negativity for the most common bullous pemphigoid target antigens: anti-BP180-NC16A-4x IgG (<2 E/mL) and anti-BP230 IgG (<2 E/mL).

**Fig 6:**
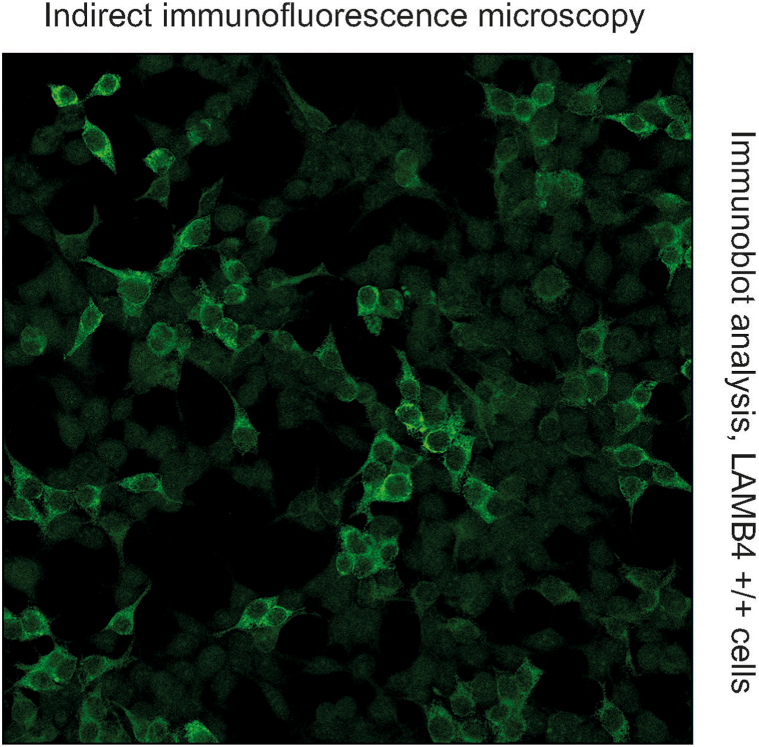
Anti-p200 pemphigoid, indirect immunofluorescence microscopy (BIOCHIP/EUROPLUS mosaics) showing an immunoblot analysis with LAMB4 positive cells.

Considering the existence of further antigens involved in the pathogenesis of the pemphigoid group diseases, the blister base reactivity without BP180/BP230 positivity prompted exploration for alternative target antigens. Immunoblot analysis using the Research Only Mosaic Dermal Binder, which employs a human cell line expressing recombinant type VII collagen, laminin 332 and laminin β4, detected strong reactivity with laminin β4 (Euroimmun, Lübeck, Germany) (Fig 6). Thus, we diagnosed anti-p200 pemphigoid, identifying laminin β4 as the target.

**Fig 7:**
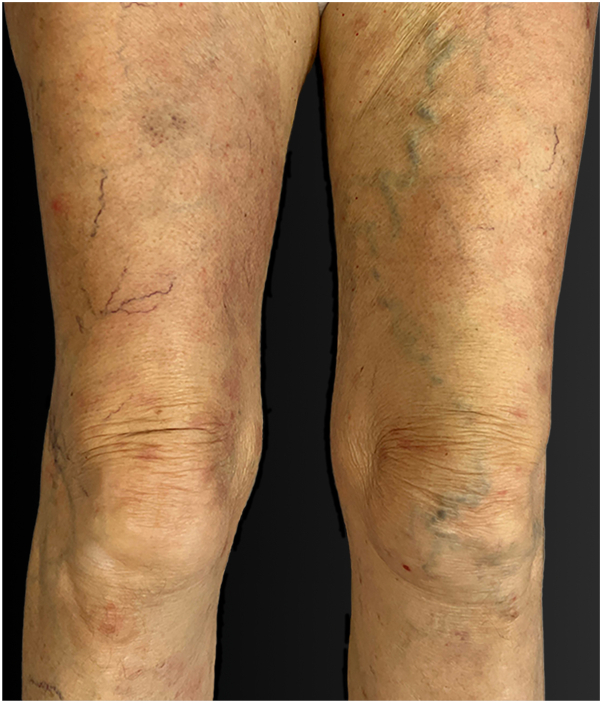
Anti-p200 pemphigoid, clinical remission after treatment. Legs of the patient.

A topical treatment regimen involving daily whole-body application of topical corticosteroids (clobetasol propionate), blister piercing, and eosin application proved ineffective. With worsening clinical presentation, the patient was hospitalized, and after a thorough examination to exclude an underlying malignancy, systemic treatment was initiated with prednisone at a dosage of 0.5 mg/kg/body weight, along with subcutaneous methotrexate injections of 15 mg/week. This combination therapy successfully controlled the disease, preventing new blister formation and promoting healing of existing skin lesions. After a 13-day hospitalization, the patient was discharged with a tapering schedule for systemic corticosteroids (prednisone), continued topical corticosteroids, and subcutaneous methotrexate injections of 20 mg/week. This treatment led to a near-complete remission. Over the subsequent 5 months, only minor relapses were observed, characterized by small, pruritic erythematous plaques on the face, neck, scalp, and palms, along with sparse bullae (Fig 7).

## Discussion

Anti-p200 pemphigoid is a distinct subepidermal autoimmune bullous disease that is clinically often similar to bullous pemphigoid, linear IgA disease, and the inflammatory variant of epidermolysis bullosa acquisita. It is characterized by autoreactive antibodies targeting structural components of the dermoepidermal junction. First described in 1996, this condition involves blistering with IgG deposits on the dermal side of the BMZ in human salt-split skin and reactivity to a 200 kDa protein in human skin extract. Its pathogenicity is due to autoantibodies targeting 200 kDa proteins, integral components of the BMZ^2^ (Fig 3).

Laminin γ1 is found in the BMZ of the skin and reacts with 70% to 90% of sera from anti-p200 pemphigoid patients. However, its specific role in the disease's pathogenicity remains disputed.^3^^,^^4^

Recently, the β4 subunit of the laminin in the basal membrane has been identified as an additional target antigen.^4^ Laminin β4 is a newly discovered type of laminin expressed in the BMZ of several anatomical sites. The resulting clinical picture of anti-p200 pemphigoid differs from bullous pemphigoid because of its greater affinity for acral surfaces and the trunk and less so for mucosal areas such as the conjunctiva.^4^^,^^5^

Despite the importance of laminin β4 in anti-p200 pemphigoid, only one study has investigated this antigen, analyzing sera from 60 patients who showed positive reactions to recombinant laminin β4.^4^ Underdiagnosis of anti-p200 pemphigoid has been common due to the lack of a commercial diagnostic assay until recently.^3^^,^^4^ Now, an indirect IF test using recombinant laminin β4 expressed in HEK293 human cells, as applied in the present study, is widely available.

Although bullous pemphigoid is typically treated with prednisolone at 0.5 mg/kg/day or long-term use of topical corticoids,^7^ anti-p200 pemphigoid generally requires less intensive immunosuppression with lower corticoid dose, often combined with dapsone.^8^ However, in our case, disease control was achieved with a combined treatment with tapering prednisone at an initial dose of 30 mg/day and methotrexate at 15 mg/week.

Our case highlights the importance of laminin β4 in anti-p200 pemphigoid, stressing the need for comprehensive serological analysis in pemphigoid diseases. The successful management of our patient also suggests that combined prednisone and methotrexate therapy may be necessary to control anti-p200 pemphigoid.

## Conflicts of interest

Emmanuella Guenova received honoraria and/or grant support from Mallinckrodt, Helsinn, Takeda Pharmaceuticals, Recordati Rare Diseases, Novartis, Sanofi, Stemline Therapeutics, and Kyowa Kirin. The other authors have no conflicts of interest to declare.

## References

[1] Amber K.T., Murrell D.F., Schmidt E., Joly P., Borradori L. (2018). Autoimmune subepidermal bullous diseases of the skin and mucosae: clinical features, diagnosis, and management. Clinic Rev Allerg Immunol.

[2] Zillikens D., Kawahara Y., ishiko A. (1996). A novel subepidermal blistering disease with autoantibodies to a 200–kDa antigen of the basement membrane zone. J Invest Dermatol.

[3] Kridin K., Ahmed A.R. (2019). Anti-p200 pemphigoid: a systematic review. Front Immunol.

[4] Goletz S., Pigors M., Lari T.R. (2024). Laminin β4 is a constituent of the cutaneous basement membrane zone and additional autoantigen of anti-p200 pemphigoid. J Am Acad Dermatol.

[5] van Beek N., Zillikens D., Schmidt E. (2021). Bullous autoimmune dermatoses–clinical features, diagnostic evaluation, and treatment options. Dtsch Arztebl Int.

[6] Dainichi T., Kurono S., Ohyama B. (2009). Anti-laminin gamma-1 pemphigoid. Proc Natl Acad Sci U S A.

[7] Borradori L., Van Beek N., Feliciani C. (2022). Updated S2 K guidelines for the management of bullous pemphigoid initiated by the European Academy of Dermatology and Venereology (EADV). J Eur Acad Dermatol Venereol.

[8] Laufer Britva R., Amber K.T., Cohen A.D., Kridin K. (2020). Treatment and clinical outcomes in anti-p200 pemphigoid: a systematic review. J Eur Acad Dermatol Venereol.

